# Updated Progress on Group II Intron Splicing Factors in Plant Chloroplasts

**DOI:** 10.3390/cimb44090290

**Published:** 2022-09-13

**Authors:** Chu Zeng, Qingsong Jiao, Ting Jia, Xueyun Hu

**Affiliations:** 1 Jiangsu Provincial Key Laboratory of Crop Genetics and Physiology/Jiangsu Provincial Key Laboratory of Crop Genomics and Molecular Breeding/Key Laboratory of Plant Functional Genomics of the Ministry of Education, Yangzhou University, Yangzhou 225009, China; 2 Co-Innovation Center for Modern Production Technology of Grain Crops of Jiangsu Province/Joint International Research Laboratory of Agriculture and Agri-Product Safety of the Ministry of Education of China, Yangzhou University, Yangzhou 225009, China

**Keywords:** group II introns, chloroplast, splicing factors

## Abstract

Group II introns are large catalytic RNAs (ribozymes) in the bacteria and organelle genomes of several lower eukaryotes. Many critical photosynthesis-related genes in the plant chloroplast genome also contain group II introns, and their splicing is critical for chloroplast biogenesis and photosynthesis processes. The structure of chloroplast group II introns was altered during evolution, resulting in the loss of intron self-splicing. Therefore, the assistance of protein factors was required for their splicing processes. As an increasing number of studies focus on the mechanism of chloroplast intron splicing; many new nuclear-encoded splicing factors that are involved in the chloroplast intron splicing process have been reported. This report reviewed the research progress of the updated splicing factors found to be involved in the splicing of chloroplast group II introns. We discuss the main problems that remain in this research field and suggest future research directions.

## 1. Introduction

Chloroplast originates from the host’s endocytosis of cyanobacteria, meaning it retains part of the cyanobacterial genome and the gene transcription and translation system [1]. Some introns remain in the chloroplast and even spread after endosymbiosis. After the transcription of these intron-containing genes, the introns of pre-mRNAs must be removed and exons should be ligated to become mature mRNAs, which can be subsequently translated into functional proteins. The expression of chloroplast genes in plants is mainly regulated at the post-transcriptional level, including at the RNA editing, intron splicing, and translation processes [2]. The precise splicing of introns is critical for the translation of chloroplast genes, which in turn plays an important role in the functioning of chloroplasts. Splicing defects in chloroplast introns affect the assembly of photosystem complexes, which in turn affects photosynthesis, along with phenotype defects such as yellowing, albino, embryo death, and growth retardation [3,4]. Therefore, studying the chloroplast intron splicing mechanism is an important part of chloroplast gene expression and the development mechanism research.

**Figure 1:**
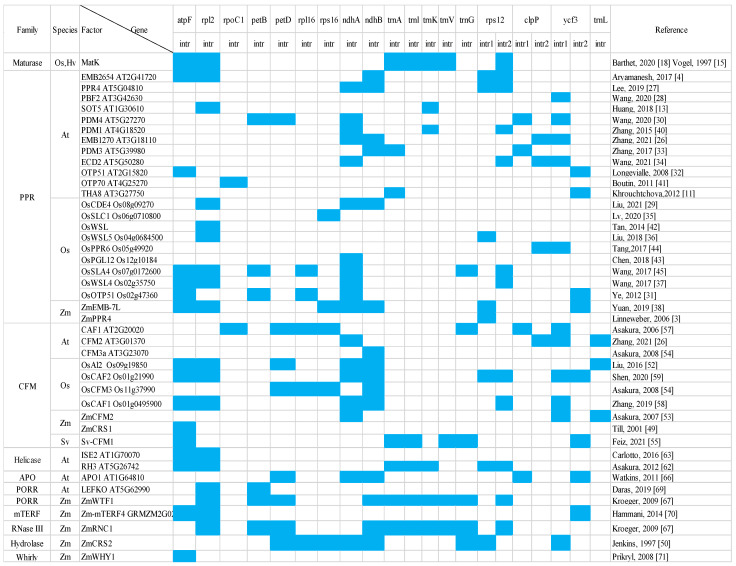
The updated splicing factors involved in chloroplast group II intron splicing. Splicing factors are listed from different protein families that are involved in chloroplast intron splicing and the corresponding references. Blank cells with blue coloring represent the splicing factors that are involved in the splicing of the introns of the indicated pre-mRNA. Os: *Oryza sativa* L.; Hv: *Hordeum vulgare* L.; At: *Arabidopsis thaliana*; Zm: *Zea mays*; Sv: *Setaria viridis*.

According to the splicing mechanism and conserved structural regions, introns in the chloroplast genome mainly include two categories: group I introns and group II introns [5]. Group II introns are further divided into the IIA and IIB subgroups based on their structural features. The main structural differences between the two intron subgroups are their exon-binding sites (EBSs), the internal loop of domain III, and the connection between domains I-VI [6,7]. The chloroplasts of higher plants contain one group I intron and nearly twenty group II introns. For example, Arabidopsis and tobacco both contain 20 group II introns, while maize and rice contain 17 [8]. Protein factors are required for the splicing of group II introns in land plant chloroplasts [9]. These proteins that assist intron splicing are referred to as splicing factors in this review. Since the first splicing factor discovery in the chloroplast made more than two decades ago, many proteins have been shown to be involved in the splicing process of one or more introns in the chloroplast [10]. These studies have provided new insights into the ribonucleic acid-protein complexes and RNA splicing mechanisms in organelles. This review mainly details the splicing factors involved in chloroplast intron splicing, specifically focusing on the recent research progress made by the literature in this field (Figure 1). We also provide suggestions for the remaining questions and future research directions in this field.

## 2. Splicing Factors of Chloroplast Group II Introns

In recent years, more and more splicing factors have been identified that are involved in chloroplast group II intron splicing. It was found that these splicing factors are mainly distributed in the pentatricopeptide repeat (PPR), chloroplast RNA splicing and ribosome maturation (CRM), RNA DEAD-box helicases, and accumulation of photosystem (APO) protein families. Members within the same family may also participate in the splicing of different introns [11,12,13]. Additionally, it has been shown that some single proteins are also involved in the splicing of chloroplast group II introns [14].

### 2.1. Maturase

Chloroplast group II introns often require the help of specific factors such as maturase for folding and efficient splicing in vivo. The only known gene encoding that uses maturase in the chloroplast of higher plants is *MATURASE K* (*matK*), which is located in the intron of the lysine *tRNA-K^UUU^* gene (*trnK)*. MatK is similar to the fungal maturation enzyme-like protein MatK [14]. It has been suggested that MatK is involved in splicing its own transcripts in vivo. Deletion of the *matK* gene product was found to be associated with the accumulation of tRNALys^UUU^-*matK* precursor transcripts in plastids, which lack functional ribosomes and mature tRNA molecules [15]. Furthermore, MatK has been shown to specifically coimmunoprecipitate with seven group IIA introns, including the introns of pre-mRNA for tRNAs (*trnA*, *trnI*, *trnV* and *trnK*), ribosomal proteins (*rpl2* and the second intron of *rps12*), and one subunit of the ATP synthase (*atpF*) [9]. The suggested MatK substrates are consistent with the reduced introns’ excision in the chloroplast ribosomal mutant, which lacked the ability for the translation of all chloroplast proteins [15,16,17]. Recently, MatK has been demonstrated to catalyze group IIA intron self-splicing for the second intron of *rps12*, but not the intron of *rpl2* in an in vitro activity assay [18]. In the future, it is worth examining the maturase activity of MatK on other group IIA introns.

### 2.2. PPR Protein

The PPR family is a large family of proteins encoded by nuclear genes that are involved in the chloroplast RNA splicing process. PPR proteins are common in most eukaryotes, especially in terrestrial plants [19]. There are more than 450 PPR genes in the Arabidopsis genome [20,21]. PPR proteins are a class of proteins that contain the PPR motif, about 35 amino acids that serve as a repeating motif. PPR proteins have been previously divided into P- and PLS-types according to the number of amino acids contained in the PPR motif [22]. The P-type PPR protein only contains the P-type of the PPR motif. PLS-like PPR proteins have three different types of PPR motifs: the P, L, and S motifs, which are arranged in tandem. PPR proteins are RNA sequence-specific binding proteins, of which almost all are located in the mitochondria or plastids, and can participate in the post-transcriptional processing of RNA, such as in the editing, splicing, and maturation processes, which is possible through its binding to specific nucleotides [20].

Many P-type PPR proteins are involved in the splicing of chloroplast group II introns. For example, HCF152 is a well-studied PPR in plants. In Arabidopsis, HCF152 binds at the site between *psbH* and *psbB* and participates in *petB* intron splicing [23]. The decreased splicing products of *petB* and reduced accumulation of the *petB* and *psbH* mRNAs in *hcf152* mutants are believed to be the direct evidence that supports HCF152’s involvement in the splicing of *petB* transcripts or its indirect effect on the degradation of mature mRNAs of *petB* and *psbH* [24]. Interestingly, a mutant allele in the conserved P residue of the C domain of HCF152 caused an impairment of *petB* splicing; however, the *psbH* 3′ and *petB* 5′ ends were almost fully protected [25]. Nakamura et al. demonstrated that the point mutation weakened the dimer formation in comparison to the wild–type HCF152 [23]. Another P-type of the PPR protein EMB1270 has become the focus of recent studies [26]. The splicing efficiencies of the *clpP1-2*, *ycf3-1*, *ndhA*, and *ndhB* introns were sharply reduced in *emb1270* mutants. An RNA immunoprecipitation (RIP)-PCR assay showed that EMB1270 specifically associated with the introns of *clpP1-2*, *ycf3-1*, *ndhA*, and *ndhB* in vivo. Moreover, an RNA electrophoretic mobility shift (REMSA) assay revealed that a truncated EMB1270 protein bound to the *clpP1-2*, *ycf3-1*, and *ndhA* introns in vitro. Finally, it was discovered that EMB1270 specifically interacted with another chloroplast splicing factor named CRM Family Member 2 (CFM2). Similar to EMB1270, several P-type PPR proteins that function as splicing factors were revealed by both genetic analysis and through their protein–RNA and/or protein–protein interaction, such as PPR4 [27], EMB2654 [4,27], PBF2 [28], OsCDE4 [29], and PDM4 [30]. However, most of the P-type PPR proteins that were found to be involved in chloroplast group II introns splicing were only revealed by comparison analyses of the abundance of pre-mRNA and spliced mature mRNA within *ppr* mutants and wild-types, such as OTP51 [31,32], PDM3 [33], ECD2 [34], OsSLC1 [35], OsWSL5 [36], OsWSL4 [37], and ZmEMB-7L [38].

Although PLS-type PPR proteins mainly act as site-specific editing factors, there are also some special PLS-type proteins that are responsible for group II intron splicing. For example, Thylakoid assembly 8 (THA8) is a member of a subfamily of plant small PPR proteins, with only four PPR motifs and not much else [11]. The splicing of *ycf3-2* and *trnA* were eliminated and strongly compromised in maize *tha8* mutants. In addition, a similar reduction of the splicing processes for *ycf3-2* and *trnA* was shown in the *tha8* mutant in Arabidopsis. ZmTHA8 coimmunoprecipitates with WTF1 and RNC1, splicing factors for *trnA* splicing [11]. In vitro gel mobility-shift assays showed that a recombination of ZmTHA8 bound five overlapping fragments of *ycf3-2*, although the binding was weak [11,39]. The crystal structures of *Brachypodium distachyon* THA8 are either free of RNA or bound to two RNA sites, revealing that RNA binding induces BdTHA8 dimerization, with a conserved G nucleotide of the bound RNAs, creating extensive contacts with both monomers [39]. PDM1/SEL1 is another PLS-type PPR protein that was found to affect the edition of *accD-1* and the splicing of group II introns in *trnK* and *ndhA* in Arabidopsis [40]. Coimmunoprecipitation mass spectrometry experiments, yeast two-hybrid, and pull-down assays have shown that PDM1 interacts with MORF9, MORF2, and MORF8, three RNA editors found in chloroplasts. In addition, an RIP assay showed that PDM1 associated with *trnK* and *ndhA*. OTP70 [41], OsWSL [42], OsPGL12 [43], OsPPR6 [44], and OsSLA4 [45] were all involved in group II intron splicing, but there is only genetic evidence through the analysis of the abundance of pre-mRNA in the related mutants. All of the known PPR proteins that were found to be involved in the splicing process of chloroplast group II introns are summarized in Figure 1.

### 2.3. CRM

The CRM domain protein originates from the prokaryotic ribosome precursor binding protein. It is homologous to the bacteria YhbY protein, and is named for its involvement in chloroplast intron splicing and the ribosome large subunit-catalyzed ribonucleoprotein assembly [46]. In eukaryotes, CRM domain proteins are only found in plants. According to their signal peptides, most of them are predicted to be localized in the plastid, and a few in the mitochondria or nuclei. The number of CRM domain proteins varies in different plants, with 16 in Arabidopsis and 14 in rice. They contain between one and four repeating CFM domains [47]. The CRM domain is an RNA recognition and binding domain, and its recognition and binding characteristics are similar to the RRM (RNA recognition motif) [48]. Studies have found that the CRM domain proteins can bind to the chloroplast intron RNA and participate in its splicing. To the best of our knowledge, six chloroplast CRM domain proteins were characterized, and they are involved in intron splicing. ZmCRS1 was the earliest defined CRM domain protein and contains three CRM domains [49,50]. The splicing of the *atpF* intron is strongly reduced in the zm*crs1* mutant [50]. CRS1 is specifically associated with the *atpF* intron in vivo, and specifically binds the *atpF* intron RNA with a high affinity in vitro [46,49,51]. There are three orthologous CRS1 proteins in rice, one of which was characterized as Albino Leaf 2 (OsAL2, Os09g19850) [52]. Surprisingly, the expression of *ndhA*, *ndhB*, *petD*, *ycf3*, and *trnL* was also significantly reduced in the *osal2* mutant, suggesting that OsCRS1 may be involved in the splicing of introns that differ from the AtCRS1 and ZmCRS1, such as the above-mentioned examples. CFM1–CFM3 are closely related paralogs with CRS1. CFM2, which harbors four CRM domains, is required for the splicing of the group I intron *trnL* and group II intron *ndhA*, *ycf3-1*, and *clpP-2* [53]. ZmCFM2 is associated with the introns of pre-*trnL-UAA*, *ndhA*, and *ycf3*, while Arabidopsis CFM2 is additionally required for the splicing of *clpP-2*. Moreover, CFM2 was found in large ribonucleoprotein particle complexes that contain CAF1 and/or CAF2, another two CRM domain proteins that are required for intron splicing [53]. CFM3, a close relative of CFM2, is dual-localized to the chloroplast and mitochondria [54]. In chloroplasts, it associates with RNAs from the *petB*, *petD*, *ndhB*, *rpl16*, *rps16*, and *trnG–UCC* loci, and the genetic data reveal that CFM3 is required for their splicing [54]. Again, it was found that CFM3 is associated with CAF1, CAF2, and RNC1 in vivo. CFM1 has been very recently characterized [55]. Three chloroplast tRNAs: *trnI*, *trnV*, and *trnA* were strongly disrupted in a *Setaria viridis cfm1* mutant. An RIP assay showed that ZmCFM1 was associated with multiple group II introns, more than the genetic data revealed the introns of *trnI*, *trnV*, and *trnA*. Finally, it was found that four chloroplast splicing factors, RNC1, THA8, mTERF4 and WTF1, overlap with intron subsets of CFM1, and coimmunoprecipitates with CFM1 [55]. It may be of interest to examine whether CAF1 and CAF2 are associated with CFM3.

CAF1 and CAF2 are closely related paralogs; each contain two CRM domains. ZmCAF1 and ZmCAF2 can also interact with ZmCRS2, respectively, to form ZmCRS2–ZmCAF1 and ZmCRS2–ZmCAF2 complexes, participating in the splicing of chloroplast group II introns and regulating chloroplast development [56]. The splicing function and intron specificities of CAF1 and CAF2 are largely conserved between maize and Arabidopsis, as was revealed by an analysis of the splicing status of chloroplast introns in *caf1* and *caf2* mutants [57]. There was an exception: the Arabidopsis CAF1–CRS2 complex additionally participated in the splicing of *rpoC1* and *clpP*, which were absent in maize chloroplasts [57]. The introns of *atpF*, *rpl2*, and *rps12* could not be spliced, and the un-spliced pre-mRNAs of *ndhA*, *ndhB*, and *ycf3* increased in *oscaf1* mutants [58]. The results suggest that OsCAF1 possesses different intron subsets because of the orthologous proteins in maize and Arabidopsis. Interestingly, an analysis of the intron splicing status in *oscaf2* mutants revealed that OsCAF2 and OsCAF1 share the same intron subsets [59].

### 2.4. DEAD-Box RNA Helicases

DEAD-box RNA helicases belong to the helicase II family and contain conserved ATP-binding domains, hydrolysis domains, RNA-binding domains, and a DEAD (Asp-Glu-Ala-Asp) sequence. DEAD-box RNA helicases are ubiquitous in all eukaryotes and many prokaryotes; they are mainly involved in ATP-dependent intramolecular and intermolecular RNA structural rearrangements, as well as in the reassembly of ribonucleoprotein complexes. Some studies have also found that DEAD-box RNA helicases are involved in RNA synthesis, modification, cleavage, degradation, ribosome biosynthesis, and translation initiation [60]. About 60 DEAD-box RNA helicases were discovered in higher plants [61], but their functions are still largely unknown. The RH3 (RNA helicase 3) of maize and Arabidopsis are conserved splicing factors. Arabidopsis *rh3* mutants have shown a reduction in the splicing of *trnI*, *trnA*, *rps12-1*, *rps12-2*, and *rpl2* [62]. In addition, an RIP assay revealed that ZmRH3 associates with these introns and with the *ycf3* intron in vivo. ISE2 was demonstrated to be another splicing factor in this family. In Arabidopsis *ise2* mutants, the splicing of *rpl2*, *atpF*, *rps12*, and *clpP* is affected, and RIP assay results have suggested that AtISE2 interacts with its RNA targets in vivo [63,64].

### 2.5. APO Family

The APO family is a new gene family discovered in recent years, which exists in both monocotyledonous and dicotyledonous plants [65]. The APO gene family members contain two conserved APO motifs separated by a less-conserved spacer sequence. Arabidopsis contains four APO proteins, APO1–APO4 [65]. APO1–APO4 share much less similarity at the N-terminus than at the C-terminus, suggesting different localizations or functions. APO1 and APO2 were predicted to be localized in the chloroplast, while APO3 and APO4 were predicted to be localized in the mitochondria [66]. ZmAPO1 was found in a coimmunoprecipitate with the splicing factor CAF1 [66]. Furthermore, AtAPO1 has been found to be involved in the splicing of the chloroplast introns *petD*, *ndhB, ndhA, ycf3-int2*, and *clpP-int1* because of the decrease in the ratio of spliced to un-spliced pre-RNA in *apo1* mutants, compared with the wild-type [66]. Moreover, recombinant ZmAPO1 and AtAPO1 bind RNA with high affinity in vitro, and the binding domain is DUF794 [66]. Aside from APO1, the functions of other members of the APO gene family have not yet been reported. The molecular mechanism of how these APOs work in vivo is still not understood.

### 2.6. PORR Family

In addition to the above-mentioned splicing factors, the members of some protein families have also been reported to be involved in chloroplast RNA splicing. The plant organellar RNA recognition (PORR) protein family has been found to be indirectly involved in RNA splicing. PORR domain proteins are nuclear-encoded, RNA-binding proteins that acquire specific functions that are involved in chloroplast RNA splicing during terrestrial plant colonization. For example, ZmWTF1 is specifically involved in the splicing of *petB, petD, ndhB, rpl2, rpl16*, and *rps16* introns [67]; in addition, ZmWTF1 interacts with RNC1 to splice the *petD, petB, ndhB, rps12-int2, trnI, trnA, trnG, trnV*, and *trnK* and introns [68]. LEFKOTHEA, another nuclear-encoded protein with a PORR domain, promotes the splicing of chloroplast group II introns in Arabidopsis. The LEFKOTHEA protein is also required for *rpl2* and *petB* intron splicing [69].

### 2.7. mTERF Family

In maize chloroplasts, Zm-mTERF4, a member of the mitochondrial transcription termination factor (mTERF) protein family, is involved in the splicing of the chloroplast *trnI*, *trnA*, *rpl2*, *ndhB*, *atpF*, and *ycf3-2* introns [70]. This conclusion was supported by the genetic data and the RIP assay that was conducted in vivo [70]. In addition, Zm-mTERF4 is found in high molecular weight complexes that contain known chloroplast splicing factors, including CAF2, CFM2, CFM3, CRS1, WHY1, RNC1, THA8, and WTF1 [70]. Additionally, it was suggested that the Zm-mTERF4 ortholog in Arabidopsis plays the conserved role of RNA splicing based on the similar physiological defects of mutants [70].

### 2.8. Whirly Family

‘Whirly’ proteins comprise a plant-specific protein family whose members have been described as DNA-binding proteins. A coimmunoprecipitation assay showed that ZmWHY1 is associated with CRS1, DNA and a subset of plastid RNAs that include *atpF* transcripts [71]. Furthermore, ZmWHY1 binds RNA and DNA in vitro as well [71]. More detailed experiments are required to show whether ZmWHY1 directly binds *atpF* transcripts in vitro. The splicing of the *atpF* intron and the content of plastid ribosomes are reduced in *zmwhy1* (Whirly) mutants [71].

## 3. Conclusions

In conclusion, the splicing of group II introns in higher plant chloroplasts requires the participation of many nuclear-encoded factors and one plastid-encoded splicing factor; both play a very important role in the regulation of chloroplast gene expression. Nucleus-encoded chloroplast intron splicing factors mostly consist of RNA binding proteins, and some can participate in the splicing of multiple group II introns in chloroplasts (Figure 1). It was suggested that splicing factors usually form splicing complexes by recruiting other protein factors. For example, CRS2 participates in the splicing process by interacting with CAF1 and CAF2, and CFM2 and CFM3 form a complex with CRS2/CAF to participate in chloroplast intron splicing. The splicing of some introns involves more than ten splicing factors (Figure 1). Further studies are needed to determine whether the splicing factors involved in the splicing of the same intron are coordinated and form a large complex to splice the introns. On the other hand, the splicing of some introns has only been found to be related to a few splicing factors, and it is unknown whether there are other splicing factors involved in their splicing that have not yet been discovered, or if their splicing requires only a small number of splicing factors. Future research is required to continuously explore new chloroplast splicing factors to update the list of splicing factors of chloroplast introns. Although more and more splicing factors have been reported for chloroplast group II introns, little is known about their working mechanisms. Recently, Yan et al. systematically identified the corresponding recognition relationship between the PPR code and the four RNA bases, providing important information about how PPR proteins recognize specific RNA sequences [72]. Until now, the splicing mechanism of chloroplast introns have seemed to be very complicated due to the multiple splicing factors that are involved in the splicing of each intron (Figure 1). The splicing of different introns is regulated by different splicing factors, and these splicing factors also belong to different protein families. It is possible that the splicing complexes are specific for each intron, and therefore many different splicing complexes are required within the chloroplast. On the other hand, the splicing complexes share some of the subunits, such as CAF1. The splicing mechanism of each chloroplast group II intron can be clarified in the future by isolating and purifying various splicing complexes and investigating the physical structure of these complexes. It is, however, gratifying that previous research groups have tried to resolve the structure of the complex that is formed by a single splicing factor and RNA; for example, the physical structure of maize ZmPPR10 and the corresponding RNA complex has been uncovered [73,74].

## Data Availability

Not applicable.

## Abbreviations

APO	ACCUMULATION OF PHOTOSYSTEM
At	*Arabidopsis thaliana*
CRM	Chloroplast RNA splicing and ribosome maturation
CRS	Chloroplast RNA splicing
CRS1	Chloroplast RNA splicing 1
CRS2	Chloroplast RNA splicing 2
CAF1	CRS2 associated factor 1
CAF2	CRS2 associated factor 2
CFM	CRM family member
CFM3A	CRM Family Member 3A
CFM3	CRM Family Member 3
CFM2	CRM Family Member 2
CFM1	CRM Family Member 1
CDE4	Chlorophyll deficient-4
EBS	Exon-binding site
EMB-7L	Embryo-specific Chromosome 7L
ECD2	Early Chloroplast Development 2
EMB1270	Embryo Defective 1270
EMB2279	Embryo Defective 2279
HCF152	High Chlorophyll Fluorescence 152
ISE2	Increased Size Exclusion Limit 2
MatK	MATURASE K
mTERF	mitochondrial transcription termination factor
Os	*Oryza sativa*
OTP51	Organelle Transcript Processing 51
OTP70	Organelle Transcript Processing 70
ORF	Open reading frame
PPR	Pentatricopeptide repeat
PORR	Plant organellar RNA recognition
PBF2	Photosystem I Biogenesis Factor 2
PDM3	Pigment-defective Mutant 3
PDM4	Pigment-defective Mutant 4
PGL12	Pale-green leaf 12
RRM	RNA recognition motif
RH3	RNA helicase 3
RNC1	RNase III-domain protein
Sv	*Setaria viridis*
SEL1	Seedling Lethal 1
SLC1	Seedling lethal chlorosis 1
SLA4	Seedling lethal albino 4
THA8	Thylakoid Assembly 8
WSL5	White-stripe leaf 5
WSL	White-stripe leaf
WSL4	White-stripe leaf 4
Zm	*Zea mays*
